# Growth and Yield Responses of Cowpea to Inoculation and Phosphorus Fertilization in Different Environments

**DOI:** 10.3389/fpls.2017.00646

**Published:** 2017-05-03

**Authors:** Stephen Kyei-Boahen, Canon E. N. Savala, David Chikoye, Robert Abaidoo

**Affiliations:** ^1^International Institute of Tropical AgricultureNampula, Mozambique; ^2^International Institute of Tropical AgricultureLusaka, Zambia; ^3^Department of Theoretical & Applied Biology, Kwame Nkrumah University of Science and TechnologyKumasi, Ghana; ^4^International Institute of Tropical Agriculture, Applied Soil Microbiology UnitIbadan, Nigeria

**Keywords:** *Bradyrhizobium* spp., inoculation, phosphorus, nodulation, nitrogen fixation, protein content, *Vigna unguiculata*

## Abstract

Cowpea (*Vigna unguiculata*) is a major source of dietary protein and essential component of the cropping systems in semi-arid regions of Sub-Saharan Africa. However, yields are very low due to lack of improved cultivars, poor management practices, and limited inputs use. The objectives of this study were to assess the effects of rhizobia inoculant and P on nodulation, N accumulation and yield of two cowpea cultivars in Mozambique. Field study was conducted in three contrasting environments during the 2013/2014 and 2014/2015 seasons using randomized complete block design with four replications and four treatments. Treatments consisted of seed inoculation, application of 40 kg P_2_O_5_ ha^-1^, inoculation + P, and a non-inoculated control. The most probable number (MPN) technique was used to estimate the indigenous bradyrhizobia populations at the experimental sites. The rhizobia numbers at the sites varied from 5.27 × 10^2^ to 1.07 × 10^3^ cells g^-1^ soil. Inoculation increased nodule number by 34–76% and doubled nodule dry weight (78 to 160 mg plant^-1^). P application improved nodulation and interacted positively with the inoculant. Inoculation, P, and inoculant + P increased shoot dry weight, and shoot and grain N content across locations but increases in number of pods plant^-1^, seeds pod^-1^, and 100-seed weight were not consistent among treatments across locations. Shoot N content was consistently high for the inoculated plants and also for the inoculated + P fertilized plants, whereas the non-inoculated control plants had the lowest tissue N content. P uptake in shoot ranged from 1.72 to 3.77 g kg^-1^ and was higher for plants that received P fertilizer alone. Inoculation and P either alone or in combination consistently increased cowpea grain yield across locations with yields ranging from 1097 kg ha^-1^ for the non-inoculated control to 1674 kg ha^-1^ for the inoculant + P treatment. Grain protein concentration followed a similar trend as grain yield and ranged from 223 to 252 g kg^-1^ but a negative correlation between grain yield and protein concentration was observed. Inoculation increased net returns by $104–163 ha^-1^ over that for the control. The results demonstrate the potential of improving cowpea grain yield, quality and profitability using inoculant, although the cost-benefit for using P at the current fertilizer price is not attractive except when applied together with inoculant at low P site.

## Introduction

Cowpea [*Vigna Unguiculata* (L.) Walp] is a major grain legume grown in semi-arid regions of Sub-Saharan Africa. It is a major source of protein and a cheap source of quality protein for both rural and urban dwellers in Africa (Ajeigbe et al., 2012; Dube and Fanadzo, 2013). Cowpea leaves and green pods are consumed as vegetable and the dried grain is used in many different food preparations. Protein content of cowpea leaves range from 27 to 43% and protein concentration of the dry grain range from 21 to 33% (Ahenkora et al., 1998; Ddamulira et al., 2015; Abudulai et al., 2016). In the savannas of West Africa, cowpea is a valuable source of livestock fodder making the dual purpose cultivars very attractive to farmers (Singh et al., 2003; Kamara et al., 2012). Cowpea is also an important component of the traditional cropping systems because it fixes atmospheric nitrogen and contributes to soil fertility improvement particularly in smallholder farming systems where little or no fertilizer is used. It is drought tolerant and adapted to stressful environments where many crops fail to grow well (Bisikwa et al., 2014; Ddamulira et al., 2015). Many cultivars have short growing cycle maturing within 60 to 80 days and make them suitable for drought-prone regions. According to FAO, cowpea was grown on an estimated 12.3 million ha in Africa in 2014 with the bulk of production occurring on 10.6 million ha in West Africa, particularly in Niger, Nigeria, Burkina Faso, Mali and Senegal (Food and Agriculture Organization of the United Nations Statistics Division [FAOSTAT], 2016).

In Southern Africa, FAO statistics indicate that 678,000 ha of cowpea was harvested in 2014 in six of the 13 countries where data was available (Food and Agriculture Organization of the United Nations Statistics Division [FAOSTAT], 2016). In Mozambique, cowpea is grown on 378,000 ha (Food and Agriculture Organization of the United Nations Statistics Division [FAOSTAT], 2016) in intercrop systems primarily with maize, cassava, and sorghum. Under this system, cowpea grain yields are very low averaging 275 kg ha^-1^ (Food and Agriculture Organization of the United Nations Statistics Division [FAOSTAT], 2016) due to poor planting arrangement that lead to shading by the companion crops and low plant population (Woomer et al., 2004; Dube and Fanadzo, 2013), low soil fertility (Maria and Yost, 2006), inappropriate planting time, the use of traditional cowpea cultivars with low yielding potential, pest and disease attack and lack of inputs. The continuous cropping of the land with no external inputs is mining the soil of its nutrients and has led to progressive decline in yields. Folmer et al. (1998) estimated average nutrient depletion of 33 kg N, 6 kg P_2_O_5_, and 25 kg K_2_O per hectare per year under the current farming practices in Mozambique. Addressing food insecurity resulting from low crop yields would require changes to the traditional crop production practices and would need emphasis on sustainable intensification on the existing land. This would include growing more drought tolerant cultivars, using improved crop management practices such as time of planting and plant population, residue management, tillage and inputs, such as crop protection chemicals, mineral fertilizers, and *Rhizobium* inoculants.

Nitrogen and phosphorus are the most limiting nutrients on smallholder farms in Mozambique (Folmer et al., 1998; Maria and Yost, 2006) but due to limited availability of fertilizers in farming communities partly as a result of the poor infrastructure for marketing and the high cost if available, farmers cannot afford. In a recent survey, Ministry of Agriculture and Food Security of Mozambique reported that only 4.6 and 3.0% of farmers used chemical and organic fertilizers, respectively, in 2014 (Ministério da Agricultura e Segurança Alimentar [MASA], 2014). Consequently, most of the N required for crop productivity comes from biological nitrogen fixation in traditional cropping systems (Dakora and Keya, 1997). In this context, cowpea which is the most widely grown legume in Mozambique is a major player in sustaining the health of soils under smallholder farms. It is estimated that cowpea can fix up to 200 kg N ha^-1^ (Dakora et al., 1987; Giller, 2001; Rusinamhodzi et al., 2006; Adjei-Nsiah et al., 2008) and can leave a positive soil N balance of up to 92 kg ha^-1^ (Chikowo et al., 2004; Rusinamhodzi et al., 2006).

Until recently, it was assumed that indigenous *Bradyrhizobium* spp. that effectively nodulate cowpea was abundantly present in tropical soils (Caldwell and Vest, 1968; Singleton et al., 1992; Kimiti and Odee, 2010) and therefore inoculation was not necessary. However, recent studies in Brazil (Soares et al., 2006; Zilli et al., 2009; Almeida et al., 2010; Costa et al., 2011; Ferreira et al., 2013), Kenya (Onduru et al., 2008), and Tanzania (Nyoki and Ndakidemi, 2013, 2014) have shown that cowpea responds to inoculation. In these studies, application of bradyrhizobia inoculants improved nodulation and also increased shoot dry matter and grain yield. For example, in the study by Almeida et al. (2010), application of three inoculant strains separately increased cowpea grain yield by 29–50% compared with the non-inoculated control with no N fertilization. In the trials by both Onduru et al. (2008) and Nyoki and Ndakidemi (2013), inoculation increased nodulation, shoot dry weight, grain yields, and other growth variables. Furthermore, application of inoculants together with P increased dry matter and grain yields more than applying inoculant or P alone suggesting that cowpea growth and yield are limited by P deficiency. The importance of P in nodulation and grain yield of cowpea is well documented (Bationo et al., 2002; Carsky, 2003; Jemo et al., 2006; Singh et al., 2011; Ayodele and Oso, 2014; Abaidoo et al., 2016). However, limited information is available on the performance of cowpea with inoculant strains and P fertilization in soils containing indigenous rhizobia population. The inoculant strain should be able to compete successfully with the indigenous population for nodule sites, thus the size and effectiveness of the indigenous strain can influence inoculation response (Thies et al., 1991a; Brockwell et al., 1995; Toro, 1996). In their studies, Onduru et al. (2008) and Nyoki and Ndakidemi (2013) did not determine the size of the indigenous rhizobia population, hence little is known about native strains in those soils. Mathu et al. (2012) examined the effects of indigenous rhizobia versus inoculant strain on cowpea in the greenhouse. They estimated the indigenous rhizobia population size and nodule occupancy but did not confirm their results in the field. In this study, we estimated the number of the indigenous rhizobia population and evaluated the effects of inoculant and P on nodulation, N accumulation and yield of two cowpea cultivars in three contrasting agro-ecologies of Mozambique.

## Materials and Methods

### Site Description

Field experiment was conducted during the 2013/2014 and 2014/2015 cropping seasons at three locations in Mozambique: Nampula (15.2739° S, 39.3136° E; 364 m.a.s.l.) in Nampula province, Sussundenga (19.0885° S, 33.4800° E; 576 m.a.s.l.) in Manica province, and Ruace (15.1408° S, 36.4136° E; 649 m.a.s.l.) in Zambezia province. The fields were selected from different agro-ecologies within high, medium, and low cowpea production regions in communities where we had on-going activities. Historically, the fields are low input managed which had maize, sesame and fallow cropping history in the three seasons preceding the current study for Nampula; groundnuts; maize and maize for Ruace and maize, maize and sesame for Sussundenga. According to the FAO soil classification, the predominant soil type at the site in Nampula is Chromic Luvisols, Sussundenga is Brunic Arenosols and Ruace is Rhodic Ferralsols. Ten soil samples were randomly taken from 0 to 20 cm soil layer using a soil auger from each site 1 week before planting. The 10 samples from each site were combined into a composite sample and four subsamples of the composite from each site were taken to the laboratory for chemical and microbiological analyses (**Table 1**). The pH was determined using a high impedance voltmeter on 1:2 soil–water suspension. Total organic carbon was determined by Walkley–Black Method, total N was determined by The Kjeldahl method, P was determined by Olsen’s method and K was determined using ICP-OES after extraction with Mehlich 3. Soil subsamples for microbial assay were stored at 4°C until used.

**Table 1:** Soil properties (0–20 cm) and *Bradyrhizobium* population at the study sites.

Location	pH (1:2 H_2_0)	Total org. C (%)	Total N (%)	P (mg kg^-1^)	K (mg kg^-1^)	*Bradyrhizobium* number (cells g^-1^ soil)
Nampula	6.3	1.76	0.12	7.6	156.5	6.89 × 10^2^
Ruace	5.9	0.79	0.05	26.1	221.0	1.07 × 10^3^
Sussundenga	6.4	0.66	0.09	10.2	108.0	5.27 × 10^2^

### Estimation of MPN Determination

The populations of indigenous *Bradyrhizobium* spp. in the soils at the three sites were estimated by most probable number (MPN) technique (Vincent, 1970) using cowpea as the trap host. Seeds of cowpea cv. IT-18 were surfaced sterilized with 95% alcohol for 10 s to remove waxy substances and air bubbles. The seeds were further sterilized in 3% hydrogen peroxide for 2 to 5 min and rinsed with sterile distilled water for five to six times. The seeds were allowed to fully imbibe sterile distilled water for 2–6 h (Somasegaran and Hoben, 1994). They were pre-germinated in Petri dishes that contained moist sterile tissue and incubated at 28°C for 48 h. Upon emergence of the radicle, seedlings with straight radicles were selected and transferred aseptically to plastic growth pouches containing 50 ml N-free plant nutrient solution (Broughton and Dilworth, 1970) using forceps. The growth pouches were arranged in a wooden rack and kept at the greenhouse for 1 week prior to inoculation. Ten steps, 10-fold (10^-1^ to 10^-10^) serial dilution was employed in the estimation of the total number of rhizobium in the soil samples, respectively, using saline solution (0.89% NaCl) as the diluent. Each growth pouch was inoculated with 1 ml of the diluent replicated four times changing pipette tips to prevent contamination. The plants were watered with sufficient N – free nutrient solution when required. Nodulation was assessed after 28 days based on the presence or absence of root nodules. The MPN of each bradyrhizobial population at each site was determined using the most probable number enumeration system (MPNES) (Woomer et al., 1990).

### Experimental Layout

Two cowpea cultivars: IT-18, an erect type with 65–70 days maturity duration and IT-1263, a semi-erect type with 75–80 maturity cycle released by the *Instituto de Investigação Agrária de Moçambique* (IIAM) (National Research Institute of Mozambique) were used in the experiment. Commercial *Bradyrhizobium* inoculant product containing strain USDA 3456 was obtained from MEA Ltd, Nairobi, Kenya for the study. The experimental design was randomized complete block with four replications. The treatments consisted of non-inoculated control, seed inoculation with USDA 3456, application of 40 kg P_2_O_5_ ha^-1^ as single superphosphate (SSP) and inoculant and 40 kg P_2_O_5_ ha^-1^ applied together. The treatments were arranged in a factorial combination with the two cowpea cultivars. Plots consisted of seven rows of 9 m in length, 0.75 m row-spacing and 0.2 m between plants within rows. Land preparation was accomplished by two passes with a disk harrow. The experiment in the 2013/2014 season was planted on 1 January at Nampula, 16 January at Sussundenga, and 31 January at Ruace. In the 2014/2015 season, planting was done on 21 January at Sussundenga, 26 January at Nampula and 12 February at Ruace.

**FIGURE 1:**
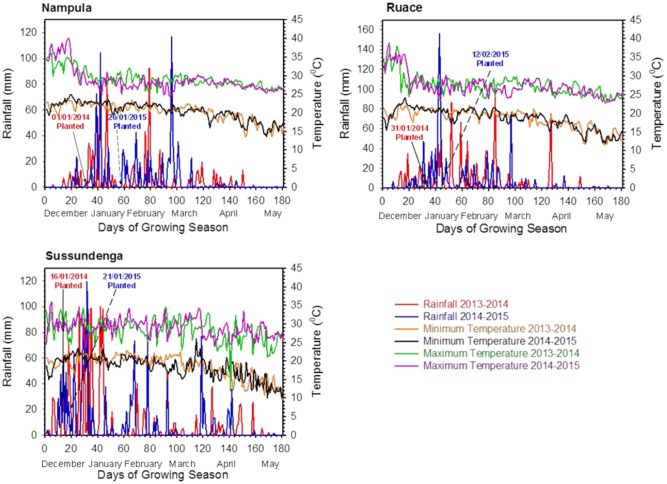
**Daily rainfall and temperature at the experimental locations during the 2013/2014 and 2014/2015 cropping seasons in Nampula, Ruace and Sussundenga, Mozambique**.

Seed inoculation was performed by weighing 0.5 kg of seeds of each cultivar into separate plastic bags and adding 2 ml of 3% (w/v) gum arabic solution as sticker. The seeds and gum arabic solution were mixed thoroughly and 5 g of the peat-based inoculant (according to the manufacturers’ recommendation) was applied to the seeds in each bag and mixed thoroughly until all the seeds were completely covered with inoculant. The inoculant was applied to supply approximately 10^6^ rhizobia cells seed^-1^. The seeds were treated in the field immediately before planting. To minimize contamination, the non-inoculated plots were planted first. Planting was done manually and weed control was done using hoe. The plants were grown under rainfed conditions (**Figure 1**). One spraying regime involving insecticide formulation consisting of 100 ml of Cypermethrin (200 g active ingredient L^-1^) + 50 ml of Lambda Cyhalomethrin (50 g active ingredient L^-1^) in 15 L water was applied with a knapsack sprayer at flowering to control insect pests.

### Data Collection

Plant stand was determined 2 weeks after seedling emergence. At flowering, five cowpea plants were randomly selected from each plot and the roots were excavated using a spade. The soil was carefully removed from the roots to ensure that the roots and the nodules were recovered as much as possible. The roots were carefully washed with water and nodules were removed and counted. The nodules were subsequently placed in envelopes and dried in an oven at 60°C for 48 h to determine nodule dry weight. The plants sampled for nodules assessment were dried in an oven at 60°C for 72 h and dry weight was determined. At maturity, 10 plants were randomly selected and pulled from the ground for pod count. The average pod number for the 10 plants was determined as the number of pods produced per plant. Forty pods from the 10 plants were randomly selected and seeds in the 40 pods were counted and the average seeds per pod were calculated. The plants from the five middle rows were harvested manually at maturity and sun-dried for about 3 days. Thereafter, pods from each plot were threshed manually and grain yield was determined. The moisture content of grain samples from each plot was determined using Farmex MT-16 grain moisture Tester (AgraTronix LLC, Streetsboro, OH, USA) and grain yield in kg ha^-1^ was calculated based on 13% moisture content. After harvesting the pods, the above-ground plant biomass in 2 m^2^ plot area in the middle of the plots were cut and sun-dried for 7 days to determined plant dry matter yield. Dried shoot and grain samples were ground to pass a 2-mm mesh sieve. Total N content was determined using the Kjeldahl method, whereas P and K were determined using ICP-OES after extraction with nitric acid and hydrochloric acid. Grain crude protein concentration was determined as total N × 6.25 (Sosulski and Imafidon, 1990). Data on cost of production and price of the grain after harvest were collected to estimate the net returns on investments. The cost estimated included the cost of seed, land preparation, planting, weeding, inoculant, P, chemical spray, harvesting, threshing, grain cleaning and bagging, transportation to selling points and other miscellaneous expenses. Except for land preparation, all field activities were done manually.

### Data Analysis

ANOVA was conducted using PROC MIXED of SAS version 9.4 (SAS Institute, 2012). Combined analysis across locations and cropping seasons was performed. Data for each location was then analyzed separately across cropping seasons. Variables analyzed include number and dry weight of nodules, shoot dry weight at flowering, shoot N, P, and K contents, number of pods per plant, number of seed per pod, 100-seed weight, grain yield, plant dry matter at harvest and grain N, P, K, and crude protein contents. However, the economic benefits of cowpea production were not analyzed statistically. The ecologies of the locations were different and had dominant effects. Moreover, the experiment was conducted under dryland conditions, thus the effects associated with the seasons were confounded with the weather that occurred in those seasons (Moore and Dixon, 2015) making seasons a random effect for the purpose of estimating variability of treatment differences for the different locations across seasons. Therefore, location, cultivar, and treatment were considered fixed effects, whereas cropping season and blocks nested within locations were considered random effects. Significant differences among treatment means were evaluated using LSD at 5% probability.

## Results

The effects of location, location × season, and location × treatment interactions influenced most of the parameters evaluated; however, the effect of location was more dominant than all the other factors. In addition, application of inoculant and P separately and together had similar effects on both cowpea cultivars across locations, and the interactions between treatment and cultivar for most of the parameters evaluated were not significant. Therefore, data for each location averaged over cultivars across the two cropping seasons are reported.

Total rainfall varied among locations and was relatively higher at Sussundenga compared to that at Ruace and Nampula (**Figure 1**). The total rainfall during the growing period at Sussundenga was 1433 and 1395 mm for 2013/2014 and 2014/2015 cropping season, respectively, whereas in Nampula the total rainfall was 827 and 997 mm, respectively; and 1084 and 1009 mm, respectively for Ruace. Except for Nampula, the total rainfall did not differ considerably between cropping seasons. Most of the rainfall in Nampula and Ruace occurred in January and February but the peak of the rain in Sussundenga was in December and January. The high rainfall at Sussundenga in January 2014 caused temporary water lodged conditions which affected seedling emergence and crop establishment. Temperatures at the locations during the two cropping seasons were not different but the average maximum temperatures in Nampula and Sussundenga during the trial period (January to April) across the seasons were 1.5°C higher than that for Ruace (**Figure 1**). On the other hand, the average minimum temperatures for Ruace and Sussundenga were similar, but both were 1.7°C lower than that for Nampula. Thus, Ruace was a relatively cooler environment, Sunsundenga relatively hot during the day and cool in the night, whereas Nampula was a hot environment.

The pH across the three locations was higher than 5.5 which was suitable for cowpea growth but organic carbon content was adequate only in Nampula (1.76%), whereas the levels at Ruace and Sussundenga were very low (0.79 and 0.66%, respectively) (**Table 1**). Similarly, total N levels ranged from 0.05 and 0.12%, indicating that the soils had low capacity to supply adequate available N. Except for Ruace which had available P considered be high (26.1 mg kg^-1^ soil), the available P at Nampula (7.6 mg kg^-1^), and Sussundenga (10.2 mg kg^-1^) were considered medium. In contrast the soil available K across the locations ranged from 108 mg kg^-1^ for Sussundenga to 221 mg kg^-1^ for Ruace and considered adequate for cowpea growth and development.

### Nodulation and Shoot Dry Matter Production

The soils across the three locations contained considerable numbers of indigenous *Bradyrhizobium* spp. which varied from 5.27 × 10^2^ to 1.07 × 10^3^ cells g^-1^ soil; however, the soil from Ruace contained the highest number of indigenous bradyrhizobia (**Table 1**). Despite the presence of indigenous strains, inoculation significantly increased the number and dry weight of nodules compared with that for the non-inoculated plants in Nampula and Ruace (**Table 2**). Although cultivar IT-1263 had higher number and dry weight of nodules than cultivar IT-18 at Ruace, cultivar × treatment interactions were not significant across locations (data not shown). Nodulation was not assessed at Sussundenga due to logistical reasons. All the treatments increased the number and dry weight of nodules in Nampula compared with that for the control and no significant differences occurred among the treatments (**Table 2**). For example in Nampula, inoculation alone increased nodule number by 5 (34%) and nodule dry weight by 41.3 mg (74%) which did not differ from those for the inoculant and P together or P alone. Similarly, all the treatments increased nodulation in Ruace but the treatments differed from each other. The number and dry weight of nodules were higher when inoculant and P were applied together followed by inoculation alone and then P alone.

**Table 2:** Effects of inoculation and P fertilization on number and dry weight of nodules, shoot dry weight at flowering, number of pods, seeds per pod and 100-seed weight averaged across two growing seasons and over two cowpea cultivars in Nampula and Ruace, Mozambique.

Treatment	No. Nod plant^-1^	Nod DW plant^-1^ (mg)	Shoot dry Wt. plant^-1^ (g)	No. Pods plant^-1^	Seeds pod^-1^	100-Seed wt. (g)
**Nampula**
Control	14.6^b^	56.1^b^	22.6^c^	23.4^a^	14.0^b^	14.0^a^
Inoculated	19.6^a^	97.4^a^	32.0^ab^	25.2^a^	15.0^a^	14.1^a^
Phosphorous (P)	18.3^a^	90.7^a^	32.3^a^	23.8^a^	15.0^a^	14.0^a^
Inoculated + P	19.4^a^	101.2^a^	27.8^b^	24.0^a^	15.2^a^	14.2^a^
**Ruace**
Control	8.4^d^	77.9^d^	37.2^b^	20.3^b^	11.6^a^	14.2^a^
Inoculated	14.8^b^	159.8^b^	48.8^a^	26.7^a^	12.0^a^	14.7^a^
Phosphorous (P)	11.7^c^	124.8^c^	44.0^a^	21.2^b^	11.9^a^	14.4^a^
Inoculated + P	18.4^a^	209.2^a^	47.9^a^	23.6^ab^	12.3^a^	14.8^a^

*Means within a column for each site followed by the same letter are not significantly different at *P* < 0.05 according to LSD*.

*Data for Sussundenga was not assessed due to logistical reasons*.

The cowpea cultivars did not vary in shoot dry matter production at flowering and no significant cultivar by treatment interaction occurred suggesting that the cultivars responded similarly to inoculation and P application across locations (**Table 2**). In Nampula and Ruace, inoculant and P either applied alone or together increased shoot dry matter yield compared with that for the controls plants but there were no differences in shoot dry matter production among the treatments at Ruace. Shoot dry weight at flowering increased from 22.6 g plant^-1^ for the control to 32.3 g plant^-1^ when P alone was applied in Nampula, whereas at Ruace shoot dry matter increased from 37.2 to 48.8 g plant^-1^ for the control and plants treated with only inoculant, respectively. On average, shoot dry matter production at Ruace (44.8 g kg^-1^) was 55% higher than that at Nampula (28.7 g kg^-1^).

### Shoot N, P, and K Contents

**FIGURE 2:**
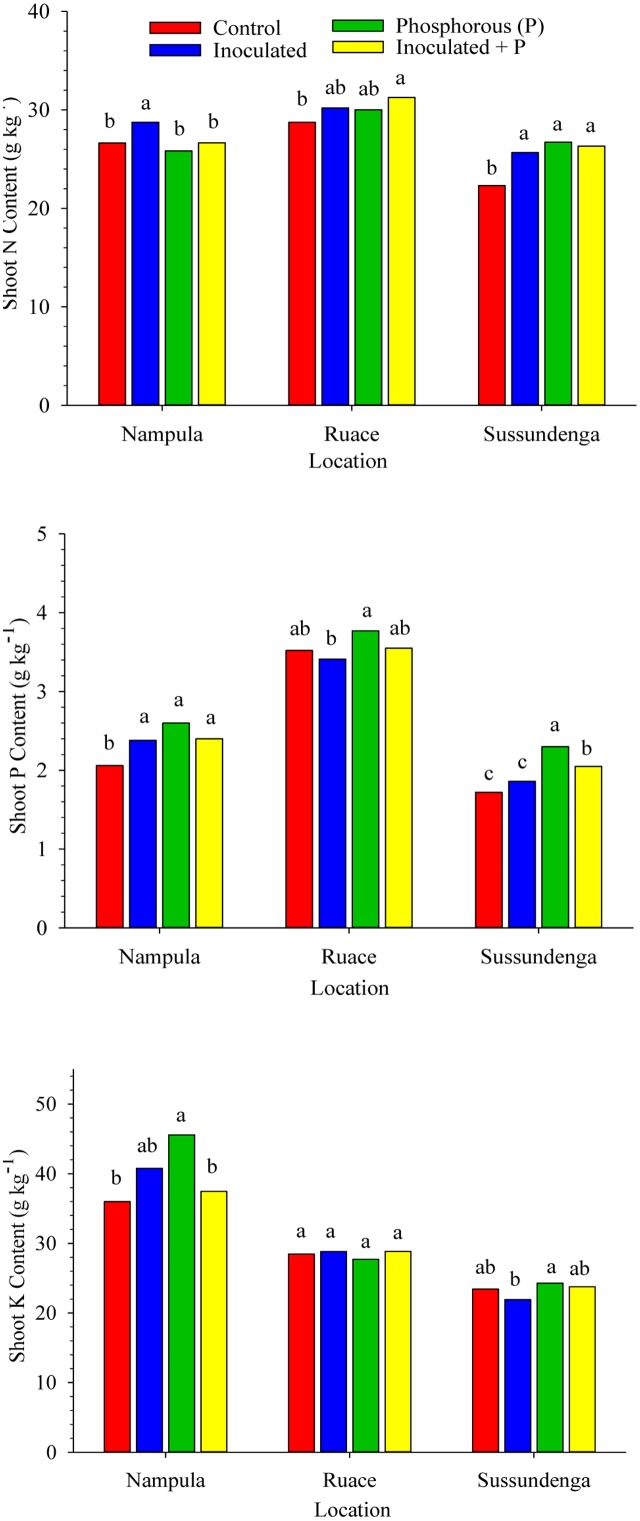
**Effects of inoculation and P fertilization on shoot N, P, and K contents averaged across two cropping seasons and over two cowpea cultivars in Nampula, Ruace and Sussundenga, Mozambique**. Means within a location followed by the same letter are not significantly different at *P* < 0.05 according to LSD.

Inoculation and P application either separately or together increased the shoot N concentration in Sussundenga but only inoculation alone increased shoot N at Nampula, whereas inoculation plus P was the only treatment that increased shoot N at Ruace (**Figure 2**). Shoot P content was consistently higher in plants that received P application although inoculation in Nampula increased shoot P content (**Figure 2**). In contrast, shoot K content was not consistently influenced by inoculation or P application. Only P fertilization alone in Nampula resulted in higher shoot K content (**Figure 2**). Across locations, shoot N content ranged from 22.3 to 31.3 g kg^-1^ and shoot P content ranged from 1.72 to 3.77 g kg^-1^ shoot dry weight. Shoot N and P accumulation was relatively higher at Ruace, whereas shoot K content was higher in Nampula.

### Yield and Yield Components

**FIGURE 3:**
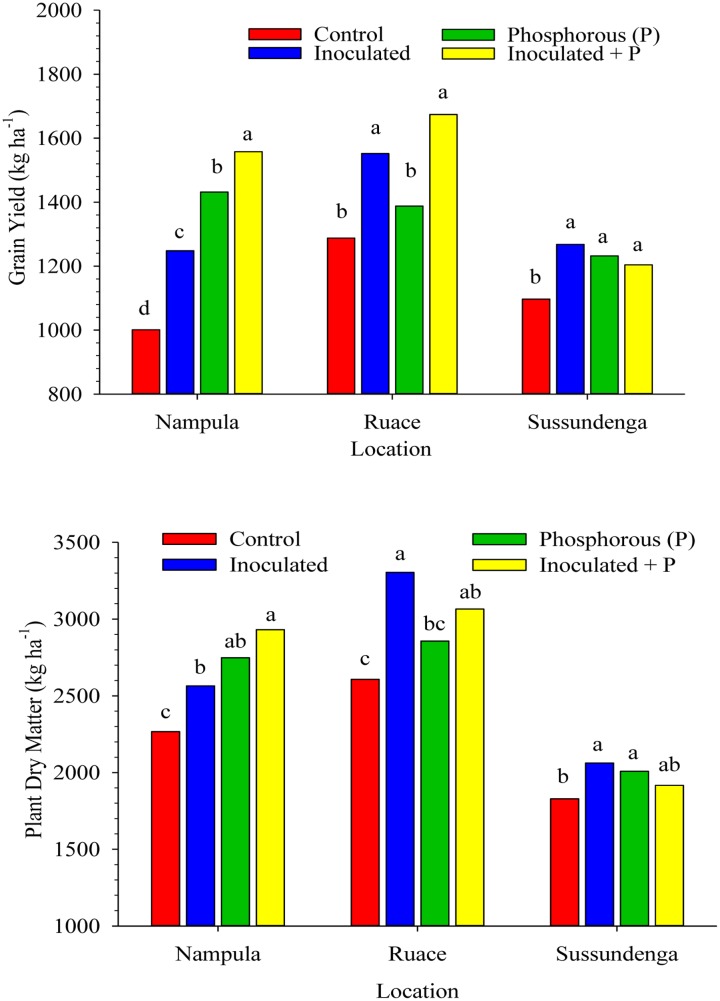
**Effects of inoculation and P fertilization on grain and dry matter yields averaged across two cropping seasons and over two cowpea cultivars in Nampula, Ruace and Sussundenga, Mozambique**. Means within a location followed by the same letter are not significantly different at *P* < 0.05 according to LSD.

Inoculation and P application increased cowpea grain yield and above-ground plant dry matter at harvest across location (**Figure 3**). Cultivar effect on grain yield and plant dry matter production as well as cultivar × treatment interactions (example; for grain yield: *P* = 0.8383 at Nampula; *P* = 0.7136 at Ruace; and *P* = 0.1336 at Sussundenga) were not significant across locations. In Nampula, grain yield for all the treatments differed from each other (**Figure 3**). Furthermore, grain yields for the treatments were higher than that for the control. Applying inoculant together with P increased grain yield by 56% (557 kg ha^-1^), P alone increased grain yield by 43% (431 kg ha^-1^) and inoculant alone improved grain yield by 25% (247 kg ha^-1^) compared with that for the non-inoculated control plants. Similarly, the above-ground dry matter yield in Nampula followed a similar trend as that for the grain yield. The order for the dry matter yield was as follows: inoculant + P > P alone > inoculant alone > non-inoculated control, although dry matter yields for P or inoculant applied separately were not different (**Figure 3**). At Ruace, inoculation alone and inoculation together with P application increased cowpea grain yield and above-ground dry matter production by an average of 325 kg ha^-1^ (25%) and 577 kg ha^-1^ (22%), respectively (**Figure 3**). Applying P alone did not affect grain and above-ground dry matter yields. At Sussundenga, applying inoculant and P either separately or in combination increased grain yield compared with that for the control (**Figure 3**). Dry matter yield at Sussundenga for the inoculant and P applied separately were higher than that for the control.

The effects of inoculant and P on the major yield components including number of pods per plant, seed per pod, and seed weight were not consistent across locations (**Table 2**). In Nampula, the number of pods per plant did not differ, although there was a trend favoring higher pods for the inoculated planted with or without P. At Ruace applying inoculant with no P increased pod number compared with that for the non-inoculated control plants but the number of pods was not different from that for applying both inoculant and P together. Number of seeds per pod did not differ at Ruace but in Nampula, inoculation and P applied separately or in combination resulted in higher number of seeds per pod compared with that for the control (**Table 2**). Seed size in terms of 100-seed weight did not respond to inoculation and P treatment across locations.

### Grain Constituents

**FIGURE 4:**
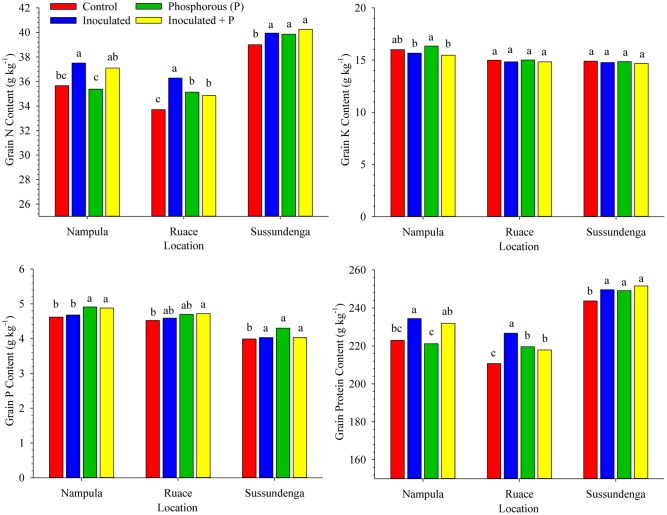
**Effects of inoculation and P fertilization on grain N, P, K, and Protein contents averaged across two cropping seasons and over two cowpea cultivars in Nampula, Ruace and Sussundenga, Mozambique**. Means within a location followed by the same letter are not significantly different at *P* < 0.05 according to LSD.

Significant differences in grain N, P, and K contents occurred among inoculation, P and control treatments. Cultivar effect and its interaction with treatment for seed composition variables were not significant except for the interaction between cultivar and treatment for grain N and for that matter protein content at Ruace (*P* < 0.0001) and Sussundenga (*P* = 0.0010). At Ruace inoculation and P either applied separately or in combination increased grain N and protein content of IT-18 compared with the control; however, grain N and protein content of IT-1263 were not affected significantly (data not shown). Similarly, grain N and protein content of IT-18 at Sussundenga did not respond to P when applied alone. In contrast, IT-1263 responded to only P when applied alone at Sussundenga, leading to significant cultivar × treatment interaction (data not shown). Averaged over cultivars, inoculation alone increased grain N and protein content in Nampula, but did not differ from that for the inoculant plus P treatment (**Figure 4**). At Ruace, all the treatments increased grain N and protein contents compared with that for the control, although inoculation alone was superior to inoculant and P applied together and P application alone. As in Ruace, inoculation, P and the combination of the two increased grain N and protein content at Sussundenga; however, the effects of the treatments did not differ from each other. The grain N and protein contents were relatively higher at Sussundenga, whereas that for Ruace was the lowest (**Figure 4**). For example, the protein content for Sussundenga ranged from 243.7 to 251.6 g kg^-1^, whilst that for Ruace ranged from 210.7 to 226.7 g kg^-1^. The mean grain protein content for Sussundenga was 29.8 and 20.9 g kg^-1^ higher than those for Ruace and Nampula, respectively. Similar to the shoot P content, application of P either alone or together with inoculant enhanced P concentration in the grain at Nampula and Sussundenga but the effect was minimal at Ruace (**Figure 4**). Generally, grain K content was not affected by any of the treatments across locations (**Figure 4**).

### Economic Benefits of Using Inoculant and Phosphorus Fertilizer

Analysis of the returns on investments showed that the cost of producing cowpea without applying inoculant or P when averaged across the growing seasons ranged from $258–276 ha^-1^ with associated net returns of $442.74–625.66 (**Table 3**). Applying inoculant alone increased net returns by $152.42 ha^-1^ at Nampula, $163.33 ha^-1^ at Ruace, and $104.03 ha^-1^ at Sussundenga compared with that for the control. Applying P alone increased the net returns only for Nampula by $92.46 compared with that for the control. However, the net returns for Ruace and Sussundenga decreased by $118.52 and $96.21, respectively, when P alone was applied. Similarly, the net benefit for applying both inputs together compared with either input applied separately was higher only at Nampula.

**Table 3:** Estimated production cost, revenue, and net returns for cowpea production averaged over 2014 and 2015 cropping seasons in Nampula, Ruace and Sussundenga, Mozambique.

Treatment	Nampula			Ruace			Sussundenga		
	Prod. cost (US$ ha^-1^)	Revenue (US$ ha^-1^)	Net returns (US$ ha^-1^)	Prod. cost (US$ ha^-1^)	Revenue (US$ ha^-1^)	Net returns (US$ ha^-1^)	Prod. cost (US$ ha^-1^)	Revenue (US$ ha^-1^)	Net returns (US$ ha^-1^)
Control	258.00	700.70	442.74	276.00	901.60	625.66	264.02	767.90	503.88
Inoculated	278.44	873.60	595.16	297.41	1086.40	788.99	279.69	887.60	607.91
Phosphorous (P)	467.20	1002.40	535.20	464.46	971.60	507.14	454.73	862.40	407.67
Inoculated + P	480.06	1090.60	610.54	487.31	1171.80	684.49	457.98	842.80	384.82

*Cost of inputs include: Seeds at $1.01 kg^-*1*^ = $25.25 ha^-*1*^; P fertilizer (20% P_*2*_O_*5*_) at $45.57 bag^-*1*^ of 50 kg = $182.28 ha^-*1*^; Inoculant = $5.00 ha^-*1*^; Chemical spray against pests = $25.57 ha^-*1*^*.

## Discussion

### *Bradyrhizobium* Effectiveness: Indigenous vs. Inoculant Strains

Cowpea forms nodules with a group of soil rhizobia classified as *Bradyrhizobium* spp., commonly present in many tropical soils (Thies et al., 1991b; Singleton et al., 1992; Martins et al., 2003; Abaidoo et al., 2007). The presence of large indigenous rhizobia population in these soils affects the effectiveness of introduced inoculant strains making it difficult to demonstrate yield response through inoculation (Thies et al., 1991a). Consequently, cowpea inoculation is not a common practice. The concept of adequate nodulation of cowpea by indigenous strains limited the interest in identifying effective and competitive strains to overcome the difficult challenge of inoculant strain establishment. The population density, effectiveness in forming nodules and competitive ability are the major factors that determine the degree of inoculation response (Singleton and Tavares, 1986; Thies et al., 1991a,b). Despite the relatively high indigenous rhizobia population size across the study locations (**Table 1**), inoculation with USDA strain 3456 increased the number and dry weight of nodules, shoot and grain N content as well as grain and dry matter yields of cowpea cultivars used (**Figures 2–4** and **Table 2**). Nodulation in the non-inoculated control treatment and in plots where P alone was applied suggested that the indigenous strains were effective in forming nodules, although the inoculant strain was superior.

### Symbiotic Efficiency of Inoculant Strains

The efficiency of inoculant strain in fixing nitrogen was demonstrated in the production of higher shoot dry matter at flowering, N accumulation in shoots and grain, and increase in yield components of inoculated plants relative to the non-inoculated plants (**Figures 2**, **4** and **Table 2**). The increase in these parameters cumulatively resulted in higher grain yield and dry matter production at harvest (**Figure 3**). Our data is consistent with the report by Martins et al. (2003) which showed that inoculation of cowpea increased nodulation, grain yield and grain N content, although the indigenous rhizobia populations at their experimental sites were relative smaller (10^1^ to 10^2^ cells g^-1^ soil) than those estimated for our study sites. Our study indicated 25, 20, and 16% increase in cowpea grain yield in Nampula, Ruace, and Sussundenga, respectively, when inoculant was applied. In the study by Martins et al. (2003) which involved 10 rhizobial isolates from cowpea nodules, significant increases in grain yield of up to 30% (533 to 693 kg ha^-1^) were observed. Our data is also consistent with other reports from Brazil where cowpea inoculation has gained popularity in recent years (Soares et al., 2006; Zilli et al., 2009; Costa et al., 2011; Ferreira et al., 2013). In these studies, inoculation increased grain yield from 341 to 957 kg ha^-1^ (Soares et al., 2006), 1539 to 2334 kg ha^-1^ (Zilli et al., 2009), and 955 to 1223 kg ha^-1^ (Costa et al., 2011) which are similar to the yield increases observed in our studies. In contrast, De Freita et al. (2012) found no effect of inoculation on cowpea grain yield and nitrogen fixation in Paraiba state in Brazil and attributed the lack of response to the presence of native rhizobia strains that formed efficient symbiosis with the local cowpea varieties. The data reported in several studies in Brazil provide appreciable evidence that increases in grain yield due to inoculation varied considerably depending on location, inoculation history of the sites and the rhizobia strains used. The ability of some strains to compete successfully with other strains in colonizing root nodule sites for nodule formation allows these strains to establish more efficient symbiosis than others. The competitive advantage may depend on the characteristics of the strains such as tolerance to drought, high temperature, low pH and other factors including host range compatibility. Strain specificity and host range compatibility have not been well characterized for cowpea (Martins et al., 2003). However, there is sufficient evidence from work conducted in Brazil using up to 10 rhizobia strains that some strains are more effective in establishing efficient symbiosis than others in cowpea that can lead to high N accumulation and grain yield (Martins et al., 2003; Soares et al., 2006; Zilli et al., 2009; Costa et al., 2011).

In eastern Kenya, Onduru et al. (2008) reported a 6.8% higher grain yield for inoculated cowpea plants compared with non-inoculated plants; and in northern Tanzania, Nyoki and Ndakidemi (2013) observed that cowpea inoculation increased nodulation, number of pods, and seed weight leading to 12% increase in grain yield. The number of pods per plant, seeds per pod, and 100-seed weight for the inoculated plants in our study were higher than those for the non-inoculated control plants, although they were not consistently significant across locations but all these together contributed to increase in grain yield and dry matter production. In contrast, our results are not consistent with data from a greenhouse study in Kenya with soil which contained 13.5 × 10^3^ rhizobia cells g^-1^ soil (Mathu et al., 2012). They found no effect of commercial inoculant on nodulation, dry matter yield and shoot N content due to the low competitive ability of the inoculant strain. In another study at five locations in Hawaii containing indigenous rhizobia population that ranged from 1.8 × 10^1^ to 3.6 x10^4^ rhizobia cells g^-1^ soil, cowpea yield and yield parameters did not respond to inoculation (Thies et al., 1991a). The authors concluded that the response to inoculation and the ability of the inoculant strains to compete successfully is inversely related to the indigenous population size. Furthermore, they found that as few as 50 rhizobia cells g^-1^ soil prevented inoculation response. The indigenous population size at our study locations were higher than three of the sites in this report (Thies et al., 1991a); hence, the discrepancy in the results of the two studies could be due to differences in the effectiveness or competitive abilities of the strains used in the two studies, Although we did not assess nodule occupancy of the inoculant strains in our study, there is sufficient evidence to suggest that the inoculant strain was competitive and formed efficient symbiosis because most of yield parameters including number and dry weight of nodules, shoot dry weight at flowering, shoot and grain N content and above-ground biomass at harvest, increased across locations. In addition to the characteristics of the indigenous and inoculant rhizobia, soil N (Streeter, 1988; Abaidoo et al., 1990) P availability (Giller, 2002; Vesterager et al., 2008; Kihara et al., 2010), pH (Brady et al., 1994), and climatic conditions (Zahran, 1999; Hungria and Vargas, 2000; Kunert et al., 2016) directly or indirectly influence yield response to inoculation. Therefore, these factors could explain the differences in the results of the various studies.

### Effects of Phosphorus and Inoculant on Cowpea Yield

Our data indicated that soil P levels limited the ability of the inoculant strain and also the indigenous rhizobia population to effectively nodulate the cowpea plants. In Nampula where the soil available P was low (**Table 1**), applying inoculant together with P increased grain yield compared with inoculation or P application alone (**Figure 3**). Inoculant together with P increased grain yield by 56% compared with that for the control plants, 24% compared with inoculation alone, and 9% compared with P application alone. Applying P alone increased grain yield by 30% (431 kg ha^-1^) compared with that for the non-inoculated control without P suggesting that nitrogen fixation by the indigenous strains was limited by the low soil available P. Plant dry matter followed a similar trend as grain yield in Nampula. However, soil available P at Ruace was relatively high (**Table 1**); hence applying inoculant and P together resulted in yield increase of only 122 kg ha^-1^ (8%) relative to applying inoculant alone. This is consistent with the fact that applying P alone did not increase grain yield at Ruace compared with that for the non-inoculated control plants. At Sussundenga where soil available P was considered medium (**Table 1**), applying either inoculant or P alone did not differ from applying both inputs together but all three treatments produced higher grain and dry matter yields relative to the non-inoculated control plants. There was also evidence that P application boosted the effectiveness and efficiency of the indigenous population as demonstrated by the higher grain yield, dry matter production, nodulation, shoot and grain N contents across locations in the treatment involving P alone compared with the control treatment. Onduru et al. (2008) also reported similar positive interaction between inoculant and P for cowpea grain yield which led to 54% increase in grain yield compared with the yield for the control. As in Nampula, the response to P was higher than that for the inoculant when applied separately due to the low soil available P at their experimental site.

Although limited information is available on cowpea inoculation, the response of cowpea to P fertilization in semi-arid areas of Africa is well documented (Ankomah et al., 1995; Bationo et al., 2002; Kolawole et al., 2002; Nyoki and Ndakidemi, 2013; Abaidoo et al., 2016). It has been demonstrated that low soil P availability constrains nitrogen fixation and cowpea productivity. This has been attributed to the important role P plays in both nodulation, nitrogen fixation and plant growth processes through enhanced root development and root hair formation (Nielsen et al., 2001; Nziguheba et al., 2016), nodule initiation and growth and as energy source for nitrogen fixation process that has direct effect on nitrogenase activity in nodules (Israel, 1987; Gordon et al., 1997; Hogh-Jensen et al., 2002) and photosynthesis (Drevon and Hartwig, 1997; Hogh-Jensen et al., 2002). Thus, application of P fertilizer to nitrogen fixing legumes on P-deficient soils further increased nitrogen fixation, yield, and yield parameters. Plants that received P fertilization had higher shoot and grain P concentrations. However, K uptake by the cowpea plants was not consistent across sites but there was the tendency for higher shoot and grain K concentrations when P fertilizer was applied. Perhaps P application stimulated K acquisition through improved root development, although soil available K across the locations were adequate (**Table 1**).

### Effects of Rhizobium Inoculation and Phosphorus Fertilization on System Productivity and Nutrition

Cowpea is grown by smallholder farmers in Mozambique and other areas of Sub-Saharan Africa under low inputs agricultural system with little or no fertilizer application; hence biological nitrogen fixation in the traditional cropping system is of vital importance for system sustainability. The cowpea residue is typically incorporated into the soil and therefore the higher N and P content in the shoots resulting from enhanced plant growth and nitrogen fixation could provide additional residual N and P for subsequent crops (Giller, 2002). In agreement with other studies (Dekhane et al., 2011; Musa et al., 2011), inoculation and P fertilization increased crude protein content of cowpea grain which is a major advantage in terms of quality nutrition. Since cowpea is an important protein source for smallholder farmers, increase in the grain protein content would improve the quality of their diet. We observed relative differences in crude protein content among locations as reported in other studies (Ddamulira et al., 2015; Sebetha et al., 2015). The differences in crude protein content could be attributed to the effects of soil and environmental conditions on plant growth. Crude protein content of legumes tend to be higher in dry locations or seasons compared with locations or seasons with adequate rainfall (Mukhtar et al., 2010; Ddamulira et al., 2015; Sebetha et al., 2015). This often leads to negative correlation between grain yield and grain N concentration as reported by others (Williams and Nakkoul, 1983; Kyei-Boahen et al., 2002). Cowpea grain yield at Sussundenga was the lowest among the locations, whereas the crude protein content was the highest possibly due to the frequent drought spells during seed filling period, although occasional heavy rains resulted in higher total rainfall than that for Nampula and Ruace where the rainfall distribution was relatively good.

### Economic Benefits of Using Inoculant and Phosphorus Fertilizer

In addition to the potential benefits of inoculant and P application on system productivity and sustainability, the results of the present study also indicated that investment of $5 ha^-1^ on inoculant applied alone translated to 34% ($152.42 ha^-1^), 26% ($163.33 ha^-1^), and 21% ($104.03 ha^-1^) higher profit margins in Nampula, Ruace, and Sussundenga, respectively, compared with the non-inoculated control (**Table 3**). In contrast, applying P alone decreased profits by $118.52 and $96.21 ha^-1^ at Ruace and Sussundenga, respectively, due to the high cost of P fertilizer. As a result of the low soil P content in Nampula, the yield response to P was high which translated to positive net returns ($92.46 ha^-1^) but was $60 ha^-1^ lower than the profit from using inoculant alone. The cost for P fertilizer was $187.28 ha^-1^ which accounted for 39.5% of the production cost, whereas the cost of inoculant was only 1.7% of the production cost. Although, applying inoculant and P together increased net returns by $167.80 and $58.83 ha^-1^ over that for the control in Nampula and Ruace, respectively, it decreased the net returns at Sussundenga by $114.06 ha^-1^. Thus, the yield due to applying P with inoculation could not pay for the cost of the fertilizer.

## Conclusion

Cowpea responded to inoculation in soils containing indigenous Bra*dyrhizobium* populations. The effect of the inoculant strain was higher in soils with adequate available P, whereas significant response to P occurred on low P soils. Application of inoculant together with P resulted in positive interactions for most of the yield parameters and was more pronounced for yield at the low P site. The study has demonstrated that using inoculant and P can enhance food security through increased grain yield and nutritional quality of many smallholder farmers in semi-arid regions of SSA who depend largely on cowpea for their daily protein intake. Furthermore, this management practice can contribute to the sustainability of the production system by enhancing residual N and P for subsequent crops. Farmers would benefit economically from using inoculant because is not expensive (about $5 ha^-1^). However, the key constraint to the use of cowpea inoculant is the limited availability in many countries in SSA. There are commercial production facilities in Kenya and South Africa which can be distributed to farmers in Mozambique but it will require education and awareness to create sufficient demand to attract private sector involvement. In contrast, the use of phosphorus is not very attractive to many farmers due to the high cost. At the current price of P fertilizer, it would not be profitable for many farmers, especially in Ruace and Sussundenga area who consider increase in grain yield only in calculating gross margin. However, applying inoculant and P together in Nampula would be more profitable than applying inoculant alone due to the low soil P levels.

## Author Contributions

SK-B and DC designed and supervised the experiment, CS participated in field evaluation, data compilation and analysis, RA conducted the rhizobia number determination (MPN). SK-B was in charge of writing up but all authors contributed to the preparation of the manuscript.

## Conflict of Interest Statement

The authors declare that the research was conducted in the absence of any commercial or financial relationships that could be construed as a potential conflict of interest. The handling Editor declared a shared affiliation, though no other collaboration, with one of the authors RA and states that the process met the standards of a fair and objective review.
